# Extended-spectrum-β-lactamase-producing Gram-negative bacteria are associated with high mortality in children with bloodstream infections in Dar es Salaam, Tanzania

**DOI:** 10.1186/s12879-025-11629-4

**Published:** 2025-10-27

**Authors:** Sabrina J. Moyo, Joel Manyahi, Said Aboud, Kristine Mørch, Adam P. Roberts, Bjørn Blomberg, Nina Langeland

**Affiliations:** 1 Department of Clinical Science, University of Bergen, Bergen, Norway; 2 Department of Microbiology and Immunology, Muhimbili University of Health and Allied Sciences, Dar es Salaam, Tanzania; 3 Norwegian National Advisory Unit on Tropical Infectious Diseases, Haukeland University Hospital, Bergen, Norway; 4 Department of Tropical Disease Biology, Liverpool School of Tropical Medicine, Liverpool, UK; 5 National Institute for Medical Research, Dar es Salaam, Tanzania; 6 Norwegian Institute of Public Health, Oslo, Norway

**Keywords:** Bloodstream infections, Antimicrobial resistance, Extended- Spectrum-β-Lactamase, Mortality

## Abstract

**Objectives:**

Studies from Tanzania show rising prevalence of extended-spectrum β-lactamase (ESBL) amongst key bacterial pathogens. We aimed to look at the clinical impact of bloodstream infections (BSI) caused by ESBL producing Gram-negative bacteria (GNB). This study was carried out on hospitalised children aged less than five years from four hospitals of the region of Dar es Salaam.

**Methods:**

The study included 135 hospitalized children with BSI due to GNB which were screened for ESBL. Clinical outcome i.e., death or discharge, was known for 130 children of which 12% (*n* = 15) died We used univariate and multivariate regression analyses to compare the outcome i.e., in-hospital mortality and length of hospital stay of inpatients with BSI caused by ESBL- producing GNB vs. outcome of inpatients with BSI caused by non ESBL producing GNB.

**Results:**

Of the 135 children, 69 (51%) and 66 (49%) had BSI due to ESBL and non-ESBL producers respectively. Of the 15 children who died, 73% had BSI due to *Klebsiella pneumoniae* or *Escherichia coli*. Mortality among children with infections caused by ESBL and non-ESBL producers were 16% and 6%, respectively. After adjusting for differences between groups regarding age, sex, and consciousness status on admission, infection with ESBL producing bacteria was a significant predictor of mortality, OR = 29; 95% CI (1.5–540). Children infected with ESBL producing GNB had longer duration of hospital stay, OR 3.2, 95% CI (1.3–7.8). Resistance towards antimicrobial agents other than penicillin’s and cephalosporins was significantly higher among ESBL producers than non-ESBL producers e.g. gentamicin (60/69, 87%) vs. (6/66, 9.1%), OR = 67; 95% CI (22.3-198.9) and ciprofloxacin (37/69, 54%) vs. (7/66, 10.6%), OR = 8; 95%CI (3.9–24.3).

**Conclusions:**

The rapidly increasing resistance to commonly used and affordable antibiotics increases the risk of death and prolongs hospital stays in neonates in the study setting. These findings highlight the need for strengthened infection prevention and control in community settings and appropriate antibiotic use to reduce the selection and spread of ESBL-GNB in hospitals.

## Introduction

Bloodstream infection (BSI) is a frequent cause of morbidity and mortality in children. In 2019, an estimated two million out of 13.7 million infection-related deaths were due to BSIs worldwide [1]. In high income countries, diagnostic facilities are more widely available and antibiotics other than penicillin’s and cephalosporins are affordable and accessible, and BSI due to antimicrobial resistant bacteria are treated with tailored regimens based on antimicrobial susceptibility test (AST) results. In sub-Saharan Africa, where BSI is a major cause of morbidity and mortality, it is especially severe in children under five, particularly neonates [2]. The diagnostic facilities are scarce, and antimicrobials other than penicillin’s and cephalosporins are relatively expensive, and not affordable and therefore BSI treatment mostly relies on empiric treatment which includes broad spectrum antimicrobial agents. Because of a lack of blood culture and AST services, the cause of the infection remains unknown, and clinicians unwillingly often provide ineffective empiric treatment. This may result in treatment failure; prolonged hospital stays and increased mortality, together with a selective environment for antimicrobial resistance (AMR).

Studies have shown that AMR has a direct impact on the duration of illness and the outcome of a BSI. A recent report estimated that 1.27 million deaths were attributed to AMR globally and sub-Saharan Africa is the most affected region [3]. However, few studies have assessed the impact of extended spectrum beta lactamases (ESBL) producing Gram negative bacteria (GNB) on mortality in the African region [4].

Studies in Tanzania have shown increasing prevalence of ESBL producing bacteria in BSIs [5–9]. Despite the reported rising prevalence of ESBL amongst key pathogens in Tanzania, there is little information about the impact of ESBLs on clinical outcomes i.e., morbidity and mortality in patients with BSI. More than two decades ago, Blomberg et al., reported a high prevalence of paediatric BSI caused by ESBL-producing GNB at a tertiary hospital in Dar es Salaam, Tanzania, and that these infections were associated with increased mortality [8, 10]. In 2018, another report from the same tertiary hospital showed that BSI caused by multi-drug resistant (MDR) bacteria increased risk of mortality in both children and adults [11]. This report provides an update on the impact of ESBL producing bacteria responsible for BSI infection on morbidity and in-hospital mortality.

## Methods

### Study population and design

The study was a hospital-based study conducted in four major hospitals of Dar es Salaam, Tanzania from the 10th of March 2017 to 30th of July 2018, and included children aged ≤ five years of age [12]. Blood cultures were obtained from 2226 children aged ≤ 5 years who were hospitalized because of fever (≥ 37.5 °C) at three regional Referral Hospitals i.e. Amana, Temeke, and Mwananyamala, and a tertiary referral hospital, Muhimbili National Hospital (MNH). In the present study we included 135 children who had positive blood cultures with GNB. The attending physician conducted a clinical examination and documented clinical parameters, including the patient’s level of consciousness, pulse rate, respiratory rate, presence of neck stiffness, degree of irritability, and signs of jaundice. Additionally, the physician noted any antibiotics or antimalarial medications prescribed, as well as whether the patient had received such treatments prior to the collection of blood cultures [12]. Admitted patients were followed up throughout their admission until discharge or death, allowing for measurement of in-hospital mortality.

### Isolation, identification, and susceptibility testing of bacteria

One to two mL of blood was inoculated in a paediatric blood culture bottle and incubated at 35 °C using the BACTEC 9050^®^ (Becton–Dickinson, Sparks, MD, United States) culture system for a maximum of 5 days unless flagged positive earlier. Positive blood cultures were sub-cultured on sheep-blood, MacConkey, chocolate, and Sabourad Dextrose agar (SDA) plates. Standard microbiological methods such as conventional biochemical tests and analytical profile index (API) (bioMérieux SA, Marcy l’Etoile, France) were used for identifying bacterial isolates. Fungal isolates that grew on SDA were Gram stained, and for yeast cells a germ-tube test was performed. Final identification of both bacterial and fungal isolates was done with MALDI-TOF MS using the Microflex LT instrument and MALDI Biotyper 3.1 software (Bruker Daltonics, Bremen, Germany).

Antimicrobial susceptibility testing was performed on 135 GNB and was done by disk diffusion technique according to guidelines and breakpoints of the Clinical Laboratory Standards Institute [13]. We used WHONET software for laboratory AMR surveillance (https://whonet.org/software.html) to create a pattern of resistance which categorised isolates with intermediate susceptibility as resistant. Screening for ESBL producing phenotype was performed using cefotaxime and ceftazidime disk, and those with reduced susceptibility were confirmed with the BBL Sensi-Disc ESBL Confirmatory Test Disks (Becton Dickinson, Sparks, MD, United States). If the zone of inhibition around the cephalosporin/clavulanic acid disk was larger than the zone around the cephalosporin-only disk (typically by 5 mm or more), or if there’s was a clear “synergy zone” where the zones merge, the isolate was considered ESBL-positive. If there was no significant difference in the zone sizes or no synergy zone, the isolate was considered as ESBL negative. Quality control strains were included for all screening tests. These included *E. coli* ATCC 25922 and *Klebsiella pneumoniae* ATCC 700603 which was used as a control strain for a positive ESBL production.

### Statistical analysis

Statistical analyses were performed using SPSS version 28.0 (Armonk, NY: IBM Corp) and R (R Foundation for Statistical Computing, Vienna, Austria). We used death to measure in-hospital mortality and hospital length of stay as a measure for morbidity. We analysed the impact of ESBL on in-hospital mortality and length of hospital stay. In addition, we analysed the impact of receiving ineffective treatment on admission (for this study it was defined as the lack of treatment with an antibiotic which has in vitro activity against the isolated pathogen based on antimicrobial susceptibility testing results) on in-hospital mortality and length of hospital stay.

Treatment with penicillins and cephalosporins was considered in effective for all ESBL producers [13], while all other treatments (including beta-lactamase inhibitor combinations) were evaluated individually based on the in vitro susceptibility test results for each isolate. The primary exposure was phenotypic resistance status (resistant vs. susceptible any bacteria/ESBL positive GNB vs. ESBL negative GNB). To determine the attributable in-hospital mortality associated with ESBL-BSI we used a replacement scenario methodology [14, 15], where we compared outcome of children with BSI caused by ESBL- producing GNB vs. outcome of children with BSI caused by non ESBL producing GNB. Different variables were compared using either the Chi-square test or Fisher´s exact test to observe the differences of proportions. Multivariable regression analysis included age as a continuous variable, sex, study site and severity of illness on admission in the model. A p-value of < 0.05 was considered statistically significant.

## Results

### Baseline characteristics

During the study period we isolated 236 bacteria and fungi from blood of 2226 children hospitalised with fever. All 135 GNB isolated from blood cultures were screened for ESBL production. These 135 GNB were isolated from 135 children with BSI. Table 1 shows baseline and clinical characteristics of the 135 children with GNB isolated from blood cultures. The majority of the study participants (92/135, 68%) were neonates aged 0–28 days. Of the 135 GNB isolates, 69 (51%) and 66 (49%) were ESBL positive and negative, respectively. Table 1 shows the proportion of ESBL producers among different species. The highest prevalence of ESBL was seen in *Klebsiella pneumoniae* followed by *Escherichia coli*. The prevalence of blood stream infections caused by ESBL producing GNB in neonates i.e. babies < 28 days was high 90% (62/69). Patients infected with ESBL producing bacteria and non-ESBL producing bacteria were similar regarding gender. There were no statistically significant differences in state of consciousness at admission between children with infections caused by ESBL-producing bacteria and those caused by non ESBL-producing bacteria. The proportion of individuals in different study sites and age groups varied significantly based on whether their BSI is caused by ESBL-producing bacteria or by non ESBL-producing bacteria.

**Table 1:** Baseline characteristics of patients (*N* = 135) according to ESBL status

Baseline characteristics	Total	ESBL- producing	Non-ESBL- producing
		*N* = 69 (%)	*N* = 66 (%)
All patients	*N* = 135	69/135 (51)	66/135 (49%)
Age: <28 days	95	62/69 (90)	33/66 (50)
Sex: Male	84	40/69 (58)	44/66 (67)
Unconsciousness: Yes	13	6/37 (16)	7/41 (17)
Outcome: Died	15	11/67(16)	4/63 (6)
Empirical Treatment: Ineffective	74	60/69 (87)	14/66 (21)
Bacterial species			
*K. pneumoniae*	62	56/69 (81)	6/66 (9)
*E. coli*	28	10/69 (15)	18/66 (27)
*E. cloacae*	5	1/69 (1.5)	4/66 (6)
A. *baumannii*	10	2/69 (3)	8/66 (12)
Others∗	30	0 (0)	30/66 (45)

∗Others includes *Pseudomonas*,* Salmonella* spp, *Serratia marcescens* and *Pantoea* spp

Out of 135 children with BSI caused by Gram negative bacteria were screened for ESBL. The outcome of these children i.e., death or discharge alive was known for 130 children. In-hospital mortality was 12% (15/130). Among the 15 children who died 12 (80%) were neonates aged 0–28 days while three were children above 28 days. In-hospital mortality was 16% and 6% among children infected with ESBL-producing bacteria and non ESBL-producing GNB respectively (OR 29, 95% CI 1.5–540). Of the 15 patients who died, 10, three and two were from Temeke, Amana Regional Referral Hospitals, and Muhimbili National Hospital (MNH), respectively. The majority had BSI due to *K. pneumoniae* (7/15) followed by *E. coli* (4/15), *Pseudomonas* spp. (2/15), *Acinetobacter baumannii* (1/15) and *Serratia marcescens* (1/15). Out of the 15 who died, 47% (7/11) had BSI due to ESBL producing *K. pneumoniae* and 27% (4/11) had ESBL producing *E. coli*. Eleven out of 15 patients who died had BSI due to ESBL-producing GNB, and 13 patients received ineffective treatment on admission while only two of these patients later received effective treatment based on susceptibility testing results.

### Outcome measures

#### A: Mortality

Fifteen of 130 children (12%) with known outcome died. Table 2A and 2B shows the risk factors for longer hospital stays and for death. In the multivariable analyses, mortality was significantly higher among children infected with ESBL producing GNB (16%, 11/67) compared to children who were not infected with ESBL producing GNB (6%, 4/63), p-value 0.024, OR 29, 95% CI 29 (1.5–540). In the adjusted analysis, mortality was also significantly higher among children who received ineffective antibiotic treatment on admission (19%, 13/69) than those who received effective treatment (3%, 2/61) p-value 0.012, OR 7.3, 95% CI (1.5–34.7), showing that ESBL-producing GNB and ineffective antibiotic treatment were both independent risk factors for death.

**Table 2:** A: Risk factors for in-hospital mortality and longer duration of hospital stay (unadjusted analysis)

Demographic/Clinical characteristic	In-hospital mortality			Longer duration of hospital stays (≥ 8 days)		
Age in days		*N* = 130			*N* = 135	OR (95% CI)
	*N*	*n* (%)	OR (95% CI)	*N*	*n* (%)	
< 28 days	92	11(12)	1.2 (0.3–3.9)	95	27 (28)	1.1 (0.6–2.1)
≥28 days	38	4 (111)	1	40	10 (25)	1
Sex						
Male	80	10 (13)	1.3 (0.4-4.0)	84	22 (26)	1.2 (0.5–2.5)
Female	50	5 (10)	1	51	15 (29)	1
ESBL status						
Positive	67	11 (16)	2.9 (0.9–9.6)	69	26 (38)	3.0 (1.3–6.8)
Negative	63	4 (6)	1	66	11 (17)	1
Treatment						
Ineffective	69	13 (19)	6.8 (1.5–31.7)	74	23 (31)	0.7 (0.3–1.4)
Effective	61	2 (3)	1	61	14 (23)	1

Table 2B: Risk factors for in-hospital mortality and longer duration of hospital stay (adjusted analysis)						
Demographic/Clinical characteristic	In-hospital mortality			Longer duration of hospital stays (≥8 days)		
	*N*=130			*N*=135		
	*N*	*n* (%)	OR (95%CI)	*N*	*n* (%)	OR (95%CI)
ESBL status						
Positive	67	11 (16)	29 (1.5-540)	69	26 (38)	3.2 (1.3-7.8)
Negative	63	4 (6)	1	66	11 (17)	1
Treatment						
Ineffective	69	13 (19)	7.3 (1.5-34.7)	74	23 (31)	1.1 (0.4-2.9)
Effective	61	2 (3)	1	61	14 (23)	1

Multivariable regression analysis was adjusted for age as a continuous variable, sex, study site, severity of illness on admission

### B: Duration of hospital stay

As shown in Table 2B, children infected with ESBL producing bacteria had longer duration of hospital stay, with 38% staying more than a week in hospital compared to 17% of children who were not infected with ESBL producing bacteria (OR 3.2, 95% CI (1.3–7.8)). There were no significant differences in the duration of hospital stay among children who received ineffective treatment (23/74,31%) vs. children who received effective treatment (14//61, 23%) on admission.

### AMR pattern for other antimicrobial agents according to ESBL status

We analysed the resistance phenotype of what would have been treatment options for infections caused by ESBL producing bacteria. ESBL-producing isolates had high rates of resistance to all three antimicrobials tested, gentamicin (60/69, 87%), ciprofloxacin (37/69, 54%) and cotrimoxazole (66/69, 95.7%). For non -ESBL producing bacteria rate of resistance towards gentamicin, ciprofloxacin and cotrimoxazole were (6/66, 9%), (7/66, 11%) and (24/66, 36%) respectively.

## Discussions

The present study aimed to look at the clinical impact of ESBL-resistance in children with Gram-negative BSI. We previously reported a high rate of ESBL-producing bacteria in BSIs among 2226 hospitalised children in four major hospitals of Dar es Salaam, Tanzania [12]. Furthermore, we reported risk factors for in-hospital mortality i.e., age, study site, severity of disease at admission, bacteraemia status and malaria. The present study found that mortality was high (16%) among children with infections caused by ESBL producing bacteria compared to (6%) among children infected with non-ESBL producing bacteria. Previous studies from Tanzania and in sub-Saharan Africa, studies have shown high prevalence of extended-spectrum β-lactamase (ESBL) producing Gram-negative bacteria (GNB) in BSIs, ranging from 15 to 98% [4, 7, 10, 16–18]. Infections caused by ESBL-producing bacteria are difficult to treat and leave clinicians with few therapeutic choices. In low- and middle-income countries (LMICs) several factors further complicate management. First, lack of diagnostic facilities results in clinicians not being aware of whether the patient’s infection is caused by GNB exhibiting AMR. Even if culture and susceptibility testing can be performed, second-line antibiotics are often unavailable and if they are they may be unaffordable. There are few studies which have quantified the impact of ESBL-producing bacterial infections on clinical outcomes in African countries [4, 10, 17, 19]. In these studies, mortality among patients infected with ESBL producing bacteria were 71% in Tanzania [10], 45% in Malawi [19] and 100% in Ethiopia [17].

For this study, we determined the attributable in-hospital mortality associated with ESBL-producing GNB responsible for BSIs using a replacement scenario methodology [14, 15] where we compared outcome of children with BSI caused by ESBL- producing GNB vs. outcome of children with BSI caused by non ESBL-producing bacteria. The method of comparison between patients with BSIs caused by ESBL-producing bacteria, or not, assumes that every infection caused by ESBL-producing bacteria would be replaced by an infection caused by susceptible (non-ESBL-producing bacteria) if the spread of resistant pathogens was prevented [14].

After adjusting for differences between groups i.e., age, sex, and severity of illness on admission by using multivariable analysis, this study demonstrated that infection with ESBL producing GNB was a significant predictor of mortality. In addition, children infected with ESBL producing bacteria had 3.2-fold increase in duration of hospital stay. Our findings agree with previous finding from sixteen years earlier in the same study setting [10], and two other studies from South Africa and Ethiopia [17, 20]. It is worthy to note that although our findings concur with the previous findings from the same study setting, mortality due to blood stream infections caused by ESBL producing bacteria has been substantially reduced from 71% in the year 2001 [10] to 12% in the present study. This can partly be explained by the availability of alternative Antimicrobials for treating BSI caused by ESBL-producing GNB for children, such as fluoroquinolones which were not used for children 16 years ago. Poor outcomes associated with ESBL-producing GNB have also been reported from high income countries [21–24]. However, some studies have not found the association between BSI caused by ESBL-producing GNB and mortality, including a recent large prospective study conducted in sub-Saharan African countries (the MBIRA study, Mortality from Bacterial Infections Resistant to Antibiotics) [25–27]. The contradicting reports on the association between ESBL-producing GNB and mortality can partly be explained by the differences in populations studied and patient factors, such as younger infants possibly being more vulnerable, or the presence of co-morbidities [28, 29]. As shown in this study, most children who died (73%) were neonates aged below 28 days, and 65% (62/95) of neonates had infection caused by ESBL producing bacteria. Studies have previously reported that neonatal sepsis is a risk factor for mortality especially in LMICs [30–32]. However, ESBL-resistance was a significant risk factor for death in this study also in multivariate analysis adjusted for age. Furthermore, pathogen-related factors can also influence patient outcomes. Some studies have shown that not all BSIs have equal clinical impact; for example, *A. baumanni* or *K. pneumoniae* BSIs more often result in a fatal outcome than *E. coli* BSIs [33, 34]. This is also supported in the present study, where *K. pneumoniae* was not only the leading pathogen in BSI, but also the leading pathogen causing in-hospital mortality. In the present study four patients died who were not infected with ESBL producing GNB, two of them had BSI due to *Pseudomonas* spp. which were not ESBL producers. *Pseudomonas* spp. are known to be more virulent, difficult to treat and associated with high mortality and morbidity in BSI patients [35, 36]. The other two children who died were infected with *E. coli* and *S. marcescens* respectively, both children received appropriate treatment based on susceptibility results but one of them was severely ill on admission. Therefore, apart from the presence or absence of ESBL, geographical variation or age differences, the prevalence of different pathogens in BSI may play a role in the clinical outcome. For instance, this study found significant differences in the proportion of ESBL infections in different study sites of the same region. Additionally, availability, accessibility, and initiation of appropriate treatment (i.e., treatment with an antibiotic which has in vitro activity against the isolated bacteria) for ESBL producing pathogens may also determine clinical outcome of patients with serious BSI infections. In this study we recorded and reported the initial empirical treatment provided, of which for the majority of patients it was ineffective treatment.

One of the limitations of this study is that the timing of initiation and type of appropriate treatment given to the patients was not recorded. Some studies have shown that delay of initiation of appropriate treatment could impact patient’s outcome [37, 38]. In the present study, the treating physicians prescribed empiric therapy in accordance with Tanzanian treatment guidelines which designate penicillin’s with or without gentamicin as the first-line agents for BSI [39]. Cephalosporins are subsequently recommended as the second line drugs”.

Therefore, on admission most patients received ampicillin or amoxicillin/clavulanic acid or ceftriaxone, with or without gentamicin. The fact that 51% of the GNB detected were ESBL producers means treatment offered to at least half of the children was ineffective. This could explain the seven-fold increased mortality risk in patients who received ineffective treatment. Our findings concur with other studies in the same setting and in other countries where ineffective treatment or delay in initiation of effective treatment has demonstrated to increase risk of mortality among ESBL producing bacteria [10, 37, 40]. In addition, ESBL-producing GNB showed high rates of resistance to other antimicrobials available and commonly used in Tanzania such as gentamicin and ciprofloxacin. The additional antimicrobial resistance of ESBL-producing GNB towards other antimicrobial agents have been reported previously [41]. Additional antimicrobial resistance mechanisms leave limited availability of oral therapeutic options and underscore the importance of judicious antibiotic use to reduce the development of antibiotic resistance and its subsequent impacts of poor.

We acknowledge a second limitation of this study pertaining to the small sample size of the number of deaths, which results in wide confidence intervals for the associations between infections due to ESBL producing bacteria and mortality or length of hospital stay. To more effectively elucidate this issue, it is recommended that future research be conducted with larger sample sizes, particularly in sub-Saharan African countries where the prevalence of ESBL is on the rise. Despite the small sample size in this study, multivariable analysis showed that ESBL-producing GNB are associated with high mortality and longer duration of hospital stay among BSI patients.

## Conclusions

The present study demonstrated that children infected with ESBL-producing GNB have higher mortality risk and prolonged duration of hospital stay compared to children infected with non-ESBL producing GNB. Furthermore, ineffective treatment on admission was associated with increased mortality. These findings highlight the need for strengthened infection prevention and control in community settings and appropriate antibiotic use to reduce the selection and spread of ESBL producing GNB in hospitals.

## Data Availability

All data generated and analysed for this study are included in this manuscript file.

## Abbreviations

AMR: Antimicrobial Resistance
AST: Antimicrobial Susceptibility Test
BSI: Blood Stream Infections
ESBL: Extended Spectrum Beta Lactamases
GNB: Gram Negative Bacteria
LMICs: Low-middle income countries

## References

[1] Collaborators GBDAR. Global mortality associated with 33 bacterial pathogens in 2019: a systematic analysis for the global burden of disease study 2019. Lancet. 2022;400(10369):2221–48. 36423648 10.1016/S0140-6736(22)02185-7 PMC9763654

[2] Reddy EA, Shaw AV, Crump JA. Community-acquired bloodstream infections in Africa: a systematic review and meta-analysis. Lancet Infect Dis. 2010;10(6):417–32. 20510282 10.1016/S1473-3099(10)70072-4 PMC3168734

[3] Antimicrobial Resistance C. Global burden of bacterial antimicrobial resistance in 2019: a systematic analysis. Lancet. 2022;399(10325):629–55. 35065702 10.1016/S0140-6736(21)02724-0 PMC8841637

[4] Lester R, Musicha P, van Ginneken N, Dramowski A, Hamer DH, Garner P, et al. Prevalence and outcome of bloodstream infections due to third-generation cephalosporin-resistant Enterobacteriaceae in sub-Saharan Africa: a systematic review. J Antimicrob Chemother. 2020;75(3):492–507. 31742611 10.1093/jac/dkz464 PMC7021093

[5] Moyo S, Aboud S, Kasubi M, Maselle SY. Bacteria isolated from bloodstream infections at a tertiary hospital in Dar Es salaam, Tanzania–antimicrobial resistance of isolates. S Afr Med J. 2010;100(12):835–8. 21414278 10.7196/samj.4186

[6] Seni J, Mwakyoma AA, Mashuda F, Marando R, Ahmed M, DeVinney R, et al. Deciphering risk factors for blood stream infections, bacteria species and antimicrobial resistance profiles among children under five years of age in North-Western Tanzania: a multicentre study in a cascade of referral health care system. BMC Pediatr. 2019;19(1):32. 30684964 10.1186/s12887-019-1411-0 PMC6347777

[7] Christopher A, Mshana SE, Kidenya BR, Hokororo A, Morona D. Bacteremia and resistant gram-negative pathogens among under-fives in Tanzania. Ital J Pediatr. 2013;39:27. 23657136 10.1186/1824-7288-39-27 PMC3665601

[8] Blomberg B, Manji KP, Urassa WK, Tamim BS, Mwakagile DS, Jureen R, et al. Antimicrobial resistance predicts death in Tanzanian children with bloodstream infections: a prospective cohort study. BMC Infect Dis. 2007;7:43. 17519011 10.1186/1471-2334-7-43 PMC1891109

[9] Onken A, Said AK, Jorstad M, Jenum PA, Blomberg B. Prevalence and antimicrobial resistance of microbes causing bloodstream infections in Unguja, Zanzibar. PLoS ONE. 2015;10(12):e0145632. 26700032 10.1371/journal.pone.0145632 PMC4689456

[10] Blomberg B, Jureen R, Manji KP, Tamim BS, Mwakagile DS, Urassa WK, et al. High rate of fatal cases of pediatric septicemia caused by gram-negative bacteria with extended-spectrum beta-lactamases in Dar Es Salaam, Tanzania. J Clin Microbiol. 2005;43(2):745–9. 15695674 10.1128/JCM.43.2.745-749.2005 PMC548071

[11] Manyahi J, Kibwana U, Mgimba E, Majigo M. Multi-drug resistant bacteria predict mortality in bloodstream infection in a tertiary setting in Tanzania. PLoS ONE. 2020;15(3):e0220424. 32130227 10.1371/journal.pone.0220424 PMC7055912

[12] Moyo SJ, Manyahi J, Blomberg B, Tellevik MG, Masoud NS, Aboud S, et al. Bacteraemia, malaria, and case fatality among children hospitalized with fever in Dar Es salaam, Tanzania. Front Microbiol. 2020;11:2118. 33013772 10.3389/fmicb.2020.02118 PMC7511546

[13] CLSI. Performance Standards for Antimicrobial Susceptibility Testing. Wayne, PA2019.

[14] de Kraker MEA, Lipsitch M. Burden of antimicrobial resistance: compared to what?? Epidemiol Rev. 2022;43(1):53–64. 33710259 10.1093/epirev/mxab001 PMC8763122

[15] Kaier K, Frank U. In search of useful methods for measuring health and economic consequences of antimicrobial resistance. Clin Infect Dis. 2013;57(8):1220–2. 23881151 10.1093/cid/cit478

[16] Droz N, Hsia Y, Ellis S, Dramowski A, Sharland M, Basmaci R. Bacterial pathogens and resistance causing community acquired paediatric bloodstream infections in low- and middle-income countries: a systematic review and meta-analysis. Antimicrob Resist Infect Control. 2019;8:207. 31893041 10.1186/s13756-019-0673-5 PMC6937962

[17] Seboxa T, Amogne W, Abebe W, Tsegaye T, Azazh A, Hailu W, et al. High mortality from blood stream infection in Addis Ababa, Ethiopia, is due to antimicrobial resistance. PLoS ONE. 2015;10(12):e0144944. 26670718 10.1371/journal.pone.0144944 PMC4682922

[18] Egbe FN, Cowden C, Mwananyanda L, Pierre C, Mwansa J, Lukwesa Musyani C, et al. Etiology of bacterial sepsis and isolate resistance patterns in hospitalized neonates in Zambia. Pediatr Infect Dis J. 2023. 10.1097/INF.0000000000004008. 37364138 10.1097/INF.0000000000004008

[19] Lester R, Musicha P, Kawaza K, Langton J, Mango J, Mangochi H, et al. Effect of resistance to third-generation cephalosporins on morbidity and mortality from bloodstream infections in blantyre, malawi: a prospective cohort study. Lancet Microbe. 2022;3(12):e922–30. 36335953 10.1016/S2666-5247(22)00282-8 PMC9712123

[20] Dramowski A, Aiken AM, Rehman AM, Snyman Y, Reuter S, Grundmann H, et al. Mortality associated with third-generation cephalosporin resistance in Enterobacteriaceae bloodstream infections at one South African hospital. J Glob Antimicrob Resist. 2022;29:176–84. 35283332 10.1016/j.jgar.2022.03.001 PMC9200643

[21] Cosgrove SE, Kaye KS, Eliopoulous GM, Carmeli Y. Health and economic outcomes of the emergence of third-generation cephalosporin resistance in *Enterobacter* species. Arch Intern Med. 2002;162(2):185–90. 11802752 10.1001/archinte.162.2.185

[22] de Kraker ME, Wolkewitz M, Davey PG, Koller W, Berger J, Nagler J, et al. Burden of antimicrobial resistance in European hospitals: excess mortality and length of hospital stay associated with bloodstream infections due to Escherichia coli resistant to third-generation cephalosporins. J Antimicrob Chemother. 2011;66(2):398–407. 21106563 10.1093/jac/dkq412

[23] Tamma PD, Komarow L, Ge L, Garcia-Diaz J, Herc ES, Doi Y, et al. Clinical impact of ceftriaxone resistance in *Escherichia coli* bloodstream infections: a multicenter prospective cohort study. Open Forum Infect Dis. 2022;9(11):ofac572. 36381622 10.1093/ofid/ofac572 PMC9645644

[24] Tumbarello M, Sanguinetti M, Montuori E, Trecarichi EM, Posteraro B, Fiori B, et al. Predictors of mortality in patients with bloodstream infections caused by extended-spectrum-beta-lactamase-producing enterobacteriaceae: importance of inadequate initial antimicrobial treatment. Antimicrob Agents Chemother. 2007;51(6):1987–94. 17387156 10.1128/AAC.01509-06 PMC1891412

[25] Dramowski A, Ong’ayo G, Rehman AM, Whitelaw A, Labi AK, Obeng-Nkrumah N, et al. Mortality attributable to third-generation cephalosporin resistance in Gram-negative bloodstream infections in African hospitals: a multi-site retrospective study. JAC Antimicrob Resist. 2021;3(1):dlaa130. 34223079 10.1093/jacamr/dlaa130 PMC8210247

[26] Menashe G, Borer A, Yagupsky P, Peled N, Gilad J, Fraser D, et al. Clinical significance and impact on mortality of extended-spectrum beta lactamase-producing Enterobacteriaceae isolates in nosocomial bacteremia. Scand J Infect Dis. 2001;33(3):188–93. 11303808 10.1080/00365540151060806

[27] Aiken AM, Rehman AM, de Kraker MEA, Madrid L, Kebede M, Labi AK, et al. Mortality associated with third-generation cephalosporin resistance in enterobacterales bloodstream infections at eight sub-Saharan African hospitals (MBIRA): a prospective cohort study. Lancet Infect Dis. 2023. 10.1016/S1473-3099(23)00233-5. 37454672 10.1016/S1473-3099(23)00233-5 PMC7617135

[28] Russo A, Falcone M, Gutierrez-Gutierrez B, Calbo E, Almirante B, Viale PL, et al. Predictors of outcome in patients with severe sepsis or septic shock due to extended-spectrum beta-lactamase-producing Enterobacteriaceae. Int J Antimicrob Agents. 2018;52(5):577–85. 29969692 10.1016/j.ijantimicag.2018.06.018

[29] Wisplinghoff H, Bischoff T, Tallent SM, Seifert H, Wenzel RP, Edmond MB. Nosocomial bloodstream infections in US hospitals: analysis of 24,179 cases from a prospective nationwide surveillance study. Clin Infect Dis. 2004;39(3):309–17. 15306996 10.1086/421946

[30] Milton R, Gillespie D, Dyer C, Taiyari K, Carvalho MJ, Thomson K, et al. Neonatal sepsis and mortality in low-income and middle-income countries from a facility-based birth cohort: an international multisite prospective observational study. Lancet Glob Health. 2022;10(5):e661–72. 35427523 10.1016/S2214-109X(22)00043-2 PMC9023753

[31] Taylor AW, Blau DM, Bassat Q, Onyango D, Kotloff KL, Arifeen SE, et al. Initial findings from a novel population-based child mortality surveillance approach: a descriptive study. Lancet Glob Health. 2020;8(7):e909–19. 32562647 10.1016/S2214-109X(20)30205-9 PMC7303945

[32] Russell NJ, Stohr W, Plakkal N, Cook A, Berkley JA, Adhisivam B, et al. Patterns of antibiotic use, pathogens, and prediction of mortality in hospitalized neonates and young infants with sepsis: a global neonatal sepsis observational cohort study (NeoOBS). PLoS Med. 2023;20(6):e1004179. 37289666 10.1371/journal.pmed.1004179 PMC10249878

[33] Scheuerman O, Schechner V, Carmeli Y, Gutierrez-Gutierrez B, Calbo E, Almirante B, et al. Comparison of predictors and mortality between bloodstream infections caused by ESBL-producing *Escherichia coli* and ESBL-producing *Klebsiella pneumoniae*. Infect Control Hosp Epidemiol. 2018;39(6):660–7. 29618394 10.1017/ice.2018.63

[34] Stewardson AJ, Allignol A, Beyersmann J, Graves N, Schumacher M, Meyer R, et al. The health and economic burden of bloodstream infections caused by antimicrobial-susceptible and non-susceptible Enterobacteriaceae and Staphylococcus aureus in European hospitals, 2010 and 2011: a multicentre retrospective cohort study. Euro Surveill. 2016;21:33. 10.2807/1560-7917.ES.2016.21.33.30319 PMC4998424 27562950

[35] Kang CI, Kim SH, Park WB, Lee KD, Kim HB, Kim EC, et al. Clinical features and outcome of patients with community-acquired *Pseudomonas aeruginosa* bacteraemia. Clin Microbiol Infect. 2005;11(5):415–8. 15819873 10.1111/j.1469-0691.2005.01102.x

[36] Kang CI, Kim SH, Park WB, Lee KD, Kim HB, Kim EC, et al. Risk factors for antimicrobial resistance and influence of resistance on mortality in patients with bloodstream infection caused by *Pseudomonas aeruginosa*. Microb Drug Resist. 2005;11(1):68–74. 15770098 10.1089/mdr.2005.11.68

[37] Schwaber MJ, Carmeli Y. Mortality and delay in effective therapy associated with extended-spectrum beta-lactamase production in Enterobacteriaceae bacteraemia: a systematic review and meta-analysis. J Antimicrob Chemother. 2007;60(5):913–20. 17848376 10.1093/jac/dkm318

[38] Lim C, Mo Y, Teparrukkul P, Hongsuwan M, Day NPJ, Limmathurotsakul D, et al. Effect of delays in concordant antibiotic treatment on mortality in patients with Hospital-Acquired *Acinetobacter* species bacteremia: emulating a target randomized trial with a 13-year retrospective cohort. Am J Epidemiol. 2021;190(11):2395–404. 34048554 10.1093/aje/kwab158 PMC8561124

[39] MINISTRY OF HEALTH CD, GENDER EAC. STANDARD TREATMENT GUIDELINES AND NATIONAL ESSENTIAL MEDICINES sixth ed2021. p. 42.

[40] Schwaber MJ, Navon-Venezia S, Kaye KS, Ben-Ami R, Schwartz D, Carmeli Y. Clinical and economic impact of bacteremia with extended-spectrum-beta-lactamase-producing Enterobacteriaceae. Antimicrob Agents Chemother. 2006;50(4):1257–62. 16569837 10.1128/AAC.50.4.1257-1262.2006 PMC1426954

[41] Tellevik MG, Blomberg B, Kommedal O, Maselle SY, Langeland N, Moyo SJ. High prevalence of faecal carriage of ESBL-producing Enterobacteriaceae among children in Dar Es salaam, Tanzania. PLoS ONE. 2016;11(12):e0168024. 27936054 10.1371/journal.pone.0168024 PMC5148075

